# Utility of Diagnostic Imaging in the Diagnosis and Management of Schistosomiasis

**DOI:** 10.4172/2327-5073.1000142

**Published:** 2014-04-15

**Authors:** David U. Olveda, Remigio M. Olveda, Alfred K. Lam, Thao N.P. Chau, Yuesheng Li, Angelo Don Gisparil, Allen G.P. Ross

**Affiliations:** 1 Griffith Health Institute, Griffith University, Gold Coast Campus, Australia; 2 Department of Health, Research Institute for Tropical Medicine, Philippines; 3 Discipline of Public Health, Flinders University, Australia; 4 QIMR Berghofer Medical Research Institute, Australia; 5 Cardinal MRI Centre, Cardinal Santos Medical Centre, Philippines

## Abstract

Diagnosis of schistosomiasis is made by demonstration of the parasite ova in stools, urine,and biopsy specimens from affected organs, or presence of antibodies to the different stages of the parasite or antigens circulating in body fluids by serologic techniques. DNA of schistosomes can now also be detected in serum and stool specimens by molecular technique.However, these tests are unable to determine the severity of target organ pathology and resultant complications. Accurate assessment of schistosome-induced morbidities is now made with the use of imaging techniques like ultrasound (US), computed tomography (CT), and magnetic resonance imaging (MRI). US has made major contributions in the diagnosis of hepatosplenic and urinary form of disease. This imaging method provides real time results, is portable (can be carried to the bed side and the field) and is lower in cost than other imaging techniques. Typical findings in hepatosplenic schistosomiasis by US include: hyperechoic fibrotic bands along the portal vessels (Symmer’s fibrosis), reduction in the size of the right lobe, hypertrophy of the left lobe, splenomegaly, and ascites. More advanced ultrasound equipment like the colour Doppler ultrasound can characterize portal vein perfusion, a procedure that is critical for the prediction of disease prognosis and for treatment options for complicated portal hypertension. Although CT and MRI are more expensive, are hospital based, and require highly additional specially-trained personnel, they provide more accurate description of the pathology, not only in hepatosplenic and urinary forms of schistosomiasis, but also in the diagnosis of ectopic forms of the disease,particularly involving thebrain and spinal cord. MRI demonstrates better tissue differentiation and lack of exposure to ionizing radiation compared with CT.

## Introduction

Schistosomiasis, or Bilharzia, is a neglected tropical parasitic disease caused by blood flukes of the genus Schistosoma. Globally, it ranks third among the most devastating tropical diseases (after malaria and intestinal helminthiasis), and is a major cause of morbidity and mortality for developing endemic countries [1]. Five species infect humans, namely: *Schistosoma mansoni, Schistosoma japonicum, Schistosoma mekongi, Schistosoma intercalatum* and *Schistosoma haematobium. S. mansoni* is endemic in parts of South America, sub-Saharan Africa, the Middle East, and the Caribbean; *S. Haematobium* is found mostly in North Africa, parts of sub-Saharan Africa, and the Middle East; *S. japonicum* is endemic in China, Philippines and Indonesia; and *Schistosoma mekongi* is endemic along the Mekong River and certain tributaries in the lower Mekong basin^2^. Schistosoma intercalatum is a minor schistosomal species limited only to some western and central African countries [2]. Globally, *S. mansoni, S. Haematobium* and *S. japonicum* account for the majority of disease burden in humans [2-4].

The life cycles of all the five human schistosome species are broadly similar [2,5]. Humans get infected when they come in contact with fresh water contaminated by cercariae, the infective stage of the parasite.The cercariae attach to the host, penetrate the skin, and transform into schistosomulae. Schistosomulae then burrow through the dermis, penetrate a blood vessel wall, access the circulatory system, migrate to the lung capillaries, and enter the systemic circulation. Afterwards, they emerge as male-female worm pairs, and inhabit either the portal or pelvic vessels.This habitat in the mesenteric vasculature is exemplified by the four schistosome species except for *S. Haematobium* which prefers the urinary bladder venous plexus.The female begins to lay eggs within the mesenteric or pelvic vessels.The eggs are meant to pass out through the intestine or through the urinary bladder to complete the life cycle. Unfortunately, many eggs laid in the mesenteric vasculature are carried upstream to the liver via the portal vein and its branches. Due to its large size, the eggs get trapped in the pre-sinusoidal portal venules or in the walls of the intestines when they migrate downstream. Eggs deposited in pelvis venous plexus migrate towards the urinary tract and are trapped in the walls of the urinary bladder and ureter. Eggs may also be deposited in other sites like the lungs, brain, and rarely in other organs like fallopian tube, ovary, uterus, appendix, and heart. Eggs deposited in the target organs ignite granulomatous reactions leading to fibrosis and significant pathology [2,5].

## Clinical Morbidity Due To Schistosomiasis

There are two disease forms: acute and chronic schistosomiasis.The acute form happens in two stages. Stage one is classified by cercarial skin penetration which may cause dermatitis or “swimmer’s itch”. Stage two corresponds to the period of larval migration and oviposition by the female adult worms which cause serum sickness-like syndrome or “Katayama fever” and is manifested by chills, fever, headache, unproductive cough, and abdominal cramps [6].

The chronic form is due to granulomatous reactions to eggs deposited in the target organs.This leads to the deposition of fibrotic materials, causing the development of significant pathology in the affected organs.The chronic form may present clinically with gastrointestinal, hepatic, neurologic, or genitourinary symptoms [2]. Individuals chronically infected with schistosomes may develop target organ pathology that can potentially result in life threatening conditions. *S. mansoni*, *S. japonicum* and *S. mekongi* infection can causetarget organ pathology in the liver leading to a severe hepatosplenic form of the disease and consequent complications such as life threatening upper gastrointestinal haemorrhage due to ruptured oesophageal or gastric varices, or severe pancytopenia due to hypersplenism. *S. Haematobium* infection on the other hand can inducepathology in the urinary tract leading to obstruction (hydronephrosis), hematuria, and in a significant number of cases, development of urinary bladder carcinoma. S. intercalatum produce only minor lesions in the lower gastrointestinal tract. Less common pathologic lesions may occur in the lungs and central nervous system, but rarely in other organs. Eggs deposited in the lungs can lead to extensive granuloma formation causing corpulmonale. In the central nervous system, eggs deposited in the brain and spinal cord can cause clinical manifestations of space occupying lesions.

## Diagnosis

Schistosoma infection can be diagnosed by schistosome ova demonstration in stools or urine (gold standard) [7-10]. The ova can be demonstrated in biopsy specimens from tissue sites such as the bladder and rectal mucosa, if the eggs are not seen in faeces and urine [11]. Diagnosis can also be made by serologic tests which can detect antibodies to the different stages of the parasite or by methods that detect parasite antigens in body fluids including the blood, cerebrospinal fluid and urine [12,13]. Recent development in molecular technology is also now able to detect DNA of the different stages of schistosomes [14]. Nevertheless, these tests cannot clearly determine the severity of target organ pathology and resultant complications caused by the lesions.

Individuals chronically infected with schistosomes may develop target organ damage that can potentially result in life threatening conditions. Hence, early detection of these serious complications in endemic areas is vital.The use of clinical signs, in the evaluation of morbidity, has often been shown to be non-specific and unreliable [15-17]. More accurate assessment ofspecific organ pathology in schistosomiasis is now performed with the use of multiple imaging modalities such as ultrasound, CT scan, and MRI.

Diagnosis of schistosomiasis by imaging techniques has mostly focused on ultrasonography. Early use of grayscale ultrasound in the diagnosis of Schistosomiasis mansoni, demonstrated lesions in the liver typical of schistosome-induced fibrosis like periportal fibrosis, which appeared as echogenic tubular shadows with anechoic lumen that radiated from the portahepatis.These tubular structures when viewed crosswise appeared like “bull’s eye” lesions due to the appearance of the concentric ring of fibrosis surrounding portal venous vasculature [18-20]. Other ultrasonographic signs of schistosomiasis included hypertrophy of the left hepatic lobe, atrophy of the right hepatic lobe, gallbladder wall thickening, granulomas, and splenic nodules. Stigmata of portal venous hypertension were also demonstrated. In the diagnosis of schistosomiasis japonica liver pathology, aside from lesions similar to schistosomiasis mansoni, ultrasonography recorded mosaic or network pattern typical only for *S. japonicum* infection [18,21]. Grading of the severity of periportal fibrosis by ultrasonography in schistosomiasis mansoni has been shown to correlate with disease burden [22,23]. Due to the reliability of US, this imaging method has been routinely used in the evaluation of hepatosplenic schistosomiasis for the past 30 years [15,24-26]. US has also been found to be valuable not only in the initial diagnosis but also in monitoring the regression of pathology following treatment with anti-schistosome drugs [27-30].

**Figure 1:**
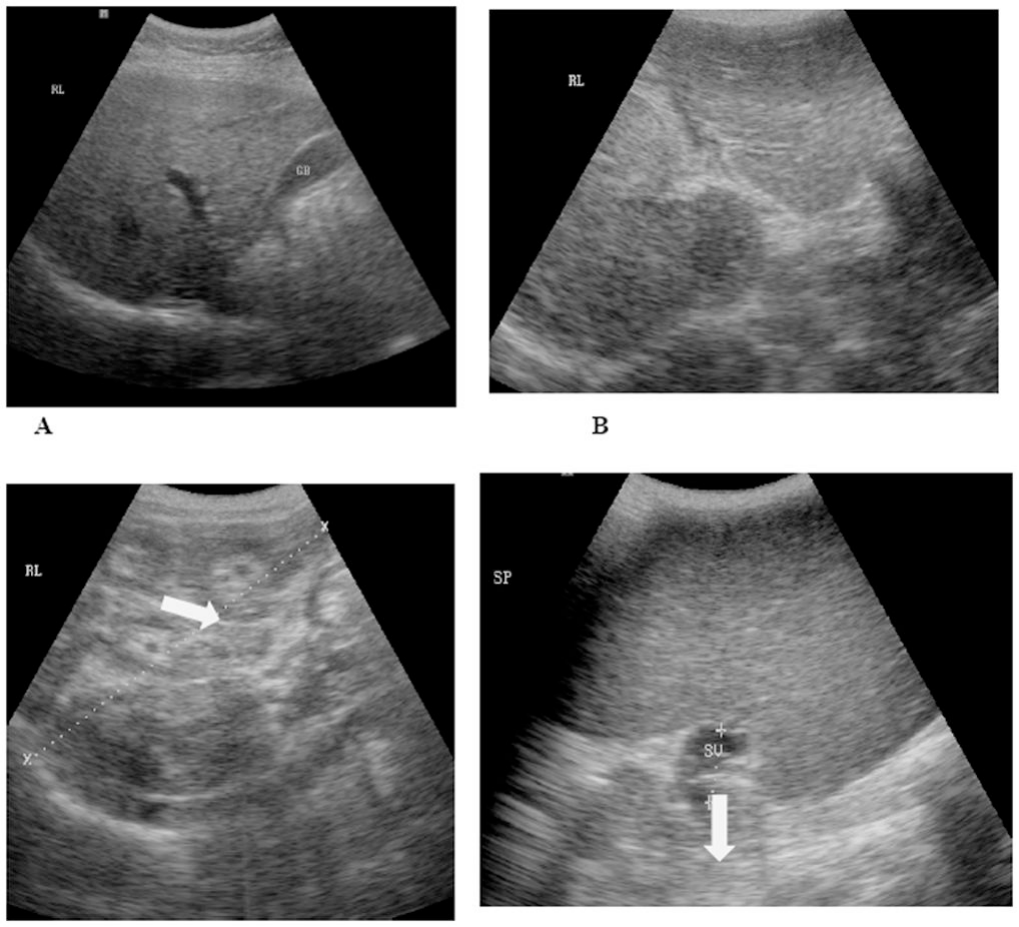
A, B, C, and D are representative ultrasound pictures that are seen in *S. japonicum* endemic areas. 1A shows a normal liver. 1B shows a liver with Grade II fibrosis (white arrow). 1C is Grade III fibrosis. The “Bull’s Eye” appearance is indicated by the white arrow in this image. Image 1D shows a very large spleen with dilated splenic vein (marked by white arrow).

**Figure 2:**
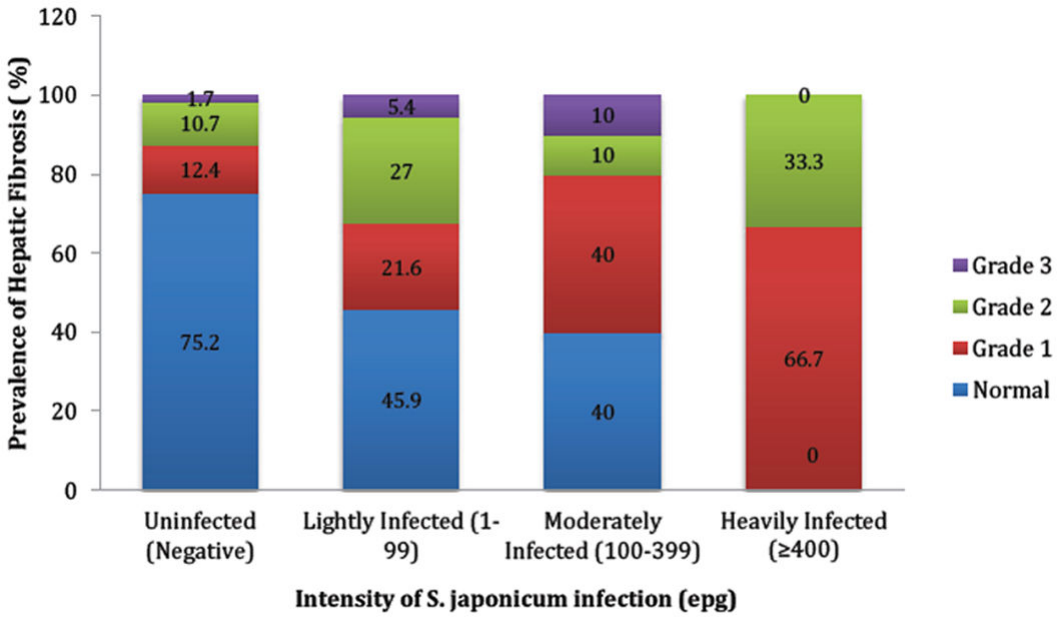
Prevalence and severity of hepatic fibrosis by *Schistosom ajaponicum* intensity in village Cabariwan. Palapag, Northern Samar, Philippines.

With the development of portable ultrasound equipment, this imaging technique became an acceptable tool in the assessment of organ morbidity due to schistosomiasis in the field setting (Appendices: Figure 1). We cite here the practical application of ultrasound at the level of endemic community.This is part of an ongoing larger study entitled, “Towards the sustainable control and elimination of schistosomiasis in the Philippines” funded by the Australian government.The purpose of this substudy was to determine the level of hepatosplenic morbidities in a village which has been the subject of mass drug administration with praziquantel for more than 20 years. One hundred seventy one (171) subjects, which represented 39% of the village population of 435, were included in the study. Males comprised 43.2% of the study population. Age specific distribution of the study population was similar to the total population.Based on the results of stool examination using Kato Katz technique 26% of the examined population were positive for *S. Japonicam* infection.Ultrasound examination was done using a portable grayscale ultrasound machine (model: SONACE). Parenchymal pattern of hepatic fibrosis was determinedaccording to the WHO guidelines for ultrasonographic examination for schistosomiasis japonica [31]. Table 1 depicts the age specific prevalence of the different degrees of fibrosis in the study population. Sixty five percent of the subjects had no fibrosis, 17% had grade I, and 18% had grade II and grade III fibrosis. Grades II and III fibrosis were considered significant fibrosis.Grade II fibrosis was first seen in age group 15-16 years old, peaked in those aged 46-55 years and then declined, thereafter. Grade III fibrosis appeared later at 36-45 age group and then increased further in the older age groups. Figure 2 shows that uninfected individuals by stool examination may have significant fibrosis. This is probably due to the limited ability of Kato Katz method to detect very light infections. On the other hand, lightly and moderately infected individuals may have no fibrosis. This could be due to fact that these individuals may have only recent infection, and it takes several years for significant fibrosis to develop.

## Ultrasound with Doppler

With the development of ultrasound with Doppler, prognostic relevance of findings seen by basic ultrasound was delineated. Doppler sonographic measurements of portal perfusion have been correlated with the presence and degree of oesophageal varices, probability of gastrointestinal bleeding, and survival in patients with cirrhosis [32-34]. Evaluation of hepatosplenic schistosomiasis japonica by Doppler sonography in the field setting, clearly showed that moderate to severe periportal thickening (PPT) or fibrosis is correlated with sonographic indicators of portal hypertension, namely reduced portal blood flow, portal systemic collaterals, dilatation of splenic vein, and splenomegaly.The authors of this studyassumed that in hepatosplenic schistosomiasis japonica, the degree of PPT, rather than the presence of network fibrosis, is a prognostic indicator for upper gastrointestinal bleeding, and thus probably for survival [35]. Patency of the portal vein can also be demonstrated by Doppler sonography. Demonstration of patent portal vein is pre-requisite to the use of transjugular intrahepatic portosystemic shunt (TIPS) in the management of complicated portal hypertension.The limitations of ultrasound with Doppler equipment are the following: it is more expensive than the ultrasound model with basic function; it has not been extensively used in field setting, and needs additional training for the ultrasonographers.

## Computerized Tomography

Computed tomography (CT) is not routinely utilized for the study and diagnosis of the schistosomiasis patient.The disadvantages of CT compared to grayscale and Doppler ultrasound would be a higher equipment and procedure cost and utilization of ionizing radiation.There is also the probability of usage of iodine-based intravenous contrast media, which not only adds to cost, but screening of patients for risk factors such as renal dysfunction and allergy history prior to administration. Like ultrasound, CT can show schistosomiasis induced changes in liver morphology, including atrophy of the right lobe and hypertrophy of the left lobe. CT depicts periportal fibrosis as a band of low attenuation around portal vein branches throughout the liver, which is enhanced following intravenous administration of contrast. Similar to the ultrasonographic findings, these enhancing periportal regions could be seen as both rounded foci and linear branching patterns, depending on the cross-sectional orientation [36].The “Bull’s Eye” lesion in the liver demonstrated by ultrasound is seen as concentric layers of periportal enhancement and is thought by some authors to be a more specific indicator of schistosomiasis rather than the periportal enhancement since this can also be associated with Kaposi sarcoma or chemotherapy [37]. Compared with ultrasound, the CT scan provides accurate diagnosis of ectopic forms of schistosomiasis involving the central nervous system, pulmonary, and other organ sites. The network pattern seen in the liver in *schistosomiasis japonica* has been delineated by CT as septal calcification [38,39]. The septal calcification of schistosomal ova in *S. japonica* has been thought to be responsible for the “turtleback” or “tortoiseshell” appearance of the hepatic lesion [39].

CT can also show splenomegaly, ascites, and prominent collateral circulation including the formation of varicose veins of the gastric fundus and the distal oesophagus, as well as, prominent collateral veins in the pelvis. CT scan has also been found to be helpful in the diagnosis of acute schistosomiasis. In some cases of acute schistosomiasis (“Katayama Syndrome”), hypodense hepatic nodules, which disappear with anti-schistosome treatment, have been demonstrated by CT [40,41].

## Magnetic Resonance Imaging

Magnetic Resonance Imaging (MRI) is a more expensive imaging modality compared both to ultrasound and CT. MRI is similar to CT in its capability of detecting ectopic forms of schistosomiasis, such as schistosomal myeloradiculopathy (SMR), the most severe and disabling ectopic form of Schistosoma mansoni infection [42,43] and cerebral schistosomiasis. Its main advantage over CT is its lack of ionizing radiation.The contrast media used for MRI is gadolinium-based, rather than the iodine-based contrast for CT.The hyperechoic periportal is seen on MRI as an accentuation of periportal signal on T2-weighted images, and hypointense signal in relation to the normal liver parenchyma in T1-weighted sequences.The periportal signal becomes accentuated on contrast enhanced T1-weighted images.It has been suggested that the hyperintense signal observed in T2-weighted sequences may differentiate periportal inflammation from fibrosis, which may not be achievable by US examination [44].

**Figure 3:**
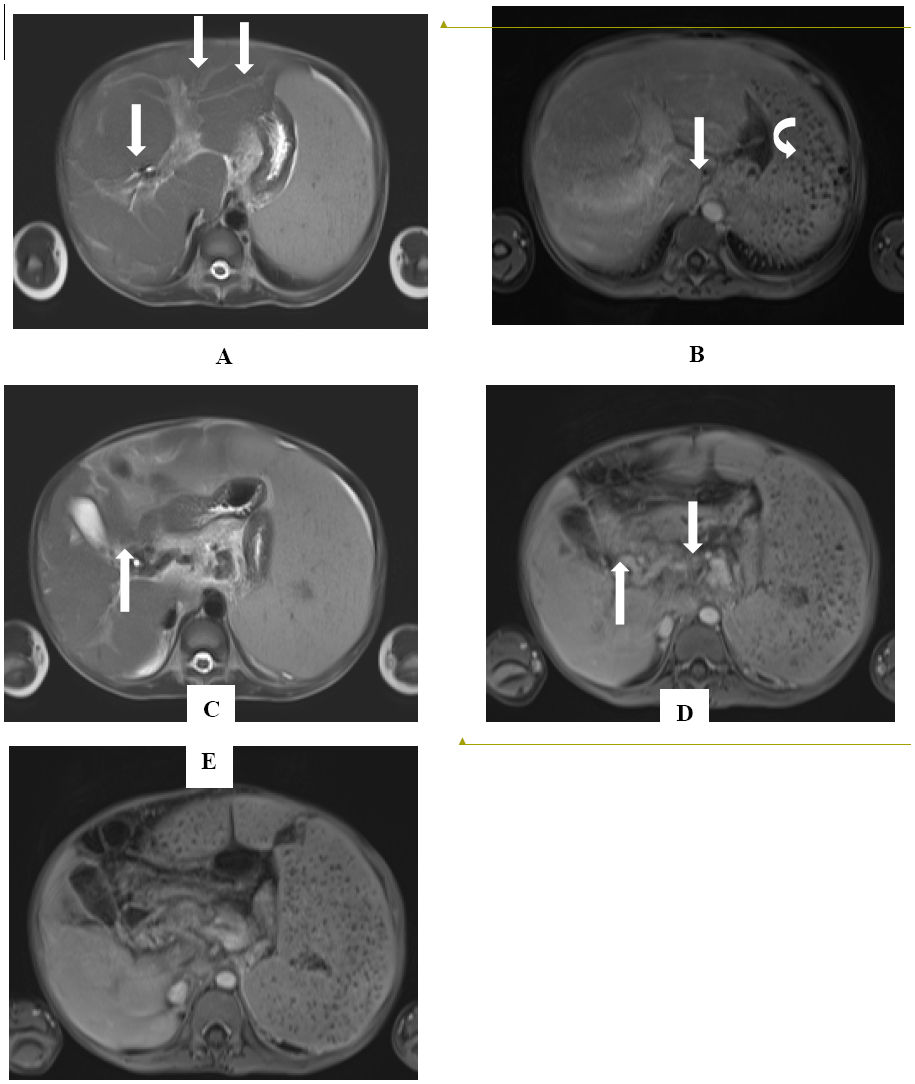
(A) Axial T2 HASTE image show hyperintense periportal fibrosis (PPF) running along the tributaries of the portal vein (arrows). (B) Axial T1 post-gadolinium VIBE image shows enhancement of the periportal fibrosis (arrows). Also seen is splenomegaly with Gamna-Gandy bodies, indicative of portal hypertension (curved arrow) (C) Axial T2 HASTE and (D) Axial T1 post-gadolinium VIBE image shows cavernous transformation of the portal vein (CTPV) showing small enhancing flow voids around the right portal vein (arrows). (E) Axial T1 post-gadolinium VIBE image shows a hypoenhancing thrombosed portal vein. (arrow).

Portal vein thrombosis (PVT), which can occur in patients with hepatosplenic schistosomiasis, is best diagnosed by MRI [45]. Cavernous transformation of portal vein (CTPV) due to PVT can be detected by colour Doppler ultrasound, CT, and MRI. Recently, we detected CTPV due to portal vein thrombosis in a 12 year old, Filipino male; with known hepatosplenic schistosomiasis japonica (MRI images are shown in Figure 3. The patient presented with rapidly enlarging spleen not compatible with the degree of fibrosis seen by ultrasound. MRI images show hypointense portal vein on post-contrast T1 weighted in Figure 3C. Formation of small vessel collaterals around the right portal vein consistent with CTPV are seen in Figure 3A and 3D.

## Conclusions

Schistosoma infection can be diagnosed by schistosome ova demonstration in stools or urine or in biopsy specimens from tissue sites of affected organs. Diagnosis is also made by serologic tests that can detect antibodies to the different stages of the parasite or detect parasite antigens in body fluids. Recently, molecular technology is able to detect DNA of the different stages of schistosomes. Nevertheless, these tests are limited to clearly determine the severity of target organ pathology and resultant complications caused by the lesions. With extensive use of antischistosome drugs, many changes are expected in the clinical manifestations of schistosomiasis. Clinical signs are non-specific and often unreliable. Different generations of imaging techniques have been developed in the last 30 years and were found to be valuable in the assessment of schistosomiasis morbidity.The major focus is on ultrasonography.

Compared with other prominent methods of medical imaging like CT and MRI, ultrasonography has several advantages. It provides images in real-time (rather than after an acquisition or processing delay), it is portable and can be brought to a sick patient’s bedside, it is substantially lower in cost, and it does not use harmful ionizing radiation. At the level of endemic communities, basic ultrasound can already provide adequate sighting of schistosome induced lesions in the liver due to *S. mansoni, S. japonicum* and *S. mekongi* and pathology in the urinary tract due to *S. haematobium*. The relevance of basic ultrasound findings have been delineated by Doppler sonogram. Doppler ultrasound can provide additional prognostic information, in addition to routine grayscale ultrasound. CT and MRI would be useful in depicting schistosomal infection in sites that may not be visualized via ultrasound. MRI has the advantage of lacking exposure to ionizing radiation compared with CT. The cost, however, is much higher than CT.

## Figures and Tables

**Table 1:** Age specific prevalence of the different degrees of fibrosis in the study population in village Cabariwan, Palapag, Northern Samar, Philippines.

Barangay Cabariwan^T^					
Fibrosis Level					
Age Group	Normal Total No (%)	Grade 1 Total No (%)	Grade 2 Total No (%)	Grade 3 Total No (%)	Total
5-15	43 (38)	5 (17)			48 (28)
16-25	12 (11)	4 (14)	2 (8)		18 (11)
26-35	20 (18)	4 (14)	1 (4)		25 (15)
36-45	10 (9)	7 (24)	7 (28)	1 (20)	25 (15)
46-55	17 (15)	6 (21)	11 (44)	2 (40)	36 (21)
>56	10 (9)	3 (10)	4 (16)	2 (40)	19 (11)
Total	112 (65)	29 (17)	25 (15)	5 (3)	171 (100)
